# Unveiling the Endocrine-Disrupting Potential of Plant-Derived Compounds: An Ecotoxicological Review

**DOI:** 10.3390/toxins17080423

**Published:** 2025-08-20

**Authors:** Changgyun Park, Heung Bin Lim

**Affiliations:** 1 Division of Experimental Neurosurgery, Department of Neurosurgery, Heidelberg University Hospital, 69120 Heidelberg, Germany; chang.park@med.uni-heidelberg.de; 2 Department of Industrial Plant Science & Technology, Chungbuk National University, Cheongju 28644, Republic of Korea

**Keywords:** plant-derived metabolites, endocrine-disrupting compounds, environmental organisms, reproductive toxicity

## Abstract

Secondary metabolites derived from plants, such as flavonoids, alkaloids, and terpenoids, are being increasingly utilized because of their bioactivity and ubiquitous distribution. Although their pharmacological uses and agricultural applications are well studied, their potential role as endocrine-disrupting compounds (EDCs) in non-target environmental organisms is largely unknown. This review aims to update our knowledge on the endocrine-disrupting effects induced by plant-derived metabolites in environmental testing models. We review guidelines and conceptual models for standardized testing approaches used to assess endocrine disruption and identify critical data gaps in the context of mammalian test systems compared to those for environmental species. We also emphasize the known endocrine mechanisms, including the regulation of estrogen and thyroid pathways and their effects on reproduction and hormonal regulation in environmental species. By integrating evidence across diverse biological systems, this work intends to provide a link between toxicological and ecological perspectives on the emerging role of plant-derived metabolites as potential EDCs in natural ecosystems. Importantly, we highlight that an extensive assessment of plant-derived metabolites is required to improve understanding of their ecological hazards and the mechanisms of their effects.

## 1. Introduction

Secondary metabolites of plant origin, such as flavonoids, alkaloids, and terpenoids, are natural compounds that play an important role in plant defense, signaling, and ecological interactions [1]. Although these compounds have been extensively researched for their therapeutic and agricultural uses, there is increasing evidence that they may cast their constituents such as quercetin and kaempferol into the environment through processes like plant decomposition, agricultural runoff, or by way of botanical pesticides or phytochemicals [2,3,4,5,6,7]. Upon introduction into the environment, such bioactive agents may encounter non-target species such as aquatic and soil biota.

Endocrine-disrupting compounds (EDCs) are exogenous agents that interfere with hormonal signaling pathways, potentially leading to adverse effects on reproduction, development, metabolism, and behavior in wildlife and humans [8]. Even though synthetic compounds, such as bisphenol A (BPA) and phthalates, have been extensively investigated with respect to EDCs, recent investigations seem to suggest that naturally produced plant metabolites can also exhibit endocrine-disrupting activities. These include estrogenic or anti-estrogenic effects, androgenic or anti-androgenic effects, aromatase activity, progesterone, modulation of thyroid hormone signaling, and interference with neuroendocrine communications [9,10,11,12,13,14,15,16]. Therefore, deciphering the endocrine-disrupting effect of natural compounds is necessary to assess their ecological risk and environmental security.

The main goal of this review is to provide a comprehensive synthesis of current knowledge on the endocrine-disrupting activity of plant-derived metabolites in environmental model organisms. For this review, we chose to study environmental species, which are frequently used in ecotoxicology. The review explores mechanisms of action, reproductive and hormonal endpoints, testing guidelines, and major data gaps for some representative natural EDCs to provide perspectives on the wider ecological impact and regulatory implications of natural EDCs.

## 2. Environmental Presence and Biological Characteristics of Plant-Derived Secondary Metabolites

### 2.1. Environmental Presence of Plant-Derived Secondary Metabolites

Secondary metabolites from plants have recently been found in a range of environmental compartments, suggesting their potential as emerging environmental contaminants. These metabolites enter natural systems through various pathways related to agricultural, pharmaceutical, and ecological practices. One major route of environmental exposure is through the use of medicinal and aromatic plants, which are cultivated or processed for bioactive compounds. During their cultivation, root exudates and leaf leachates can release flavonoids, terpenes, and alkaloids directly into the soil and nearby water bodies [6,17].

Phytoestrogens have been widely found in municipal wastewater and surface waters, primarily due to their excretion following human consumption of plant-based supplements, herbal medicines, and polyphenol-rich foods [7,18]. Wastewater treatment plants (WWTPs) serve as major entry points for these bioactive compounds into the aquatic environment. It has been extensively documented that traditional WWTPs do not completely remove phytoestrogens, which are discharged into effluents, and then into the receiving rivers or streams [7,18]. Jarošová et al. [19] further demonstrated that phytoestrogens are widely distributed in surface waters around the world and their recalcitrance raises concerns for potential endocrine-disrupting activity in aquatic organisms. Certain plant-borne compounds used as active ingredients in biopesticides, such as coumarin-based products, might also escape into the environment directly through agricultural runoff, thus presenting another route by which these active metabolites contribute to environmental contamination [20]. These routes lead to a measurable occurrence of many secondary metabolites in different types of environments. For example, genistein and daidzein—two major isoflavonoids—have been detected at high concentrations in effluents and surface waters near urban and agricultural sites [7,18,19,20]. Coumarin, a fragrance and rodenticide precursor, has been detected in surface water [19,21]. Quercetin, a common dietary flavonoid, has been detected in the influents of wastewater treatment plants at concentrations as high as 1.2 µg/L and remains present in treated effluents at levels between 50 and 300 ng/L [22]. Similarly, stevioside and rebaudioside A, commonly used as natural sweeteners, have been observed in WWTP effluent at low µg/L levels, depending on usage patterns and treatment efficiency [23].

However, as the majority of monitoring studies have targeted concentrations within the potential exposure range of humans or their influence in wastewater ecology, these results provide evidence that environmental species may be chronically exposed to such metabolites as well. Aquatic organisms, such as fish, daphnids, ostracods, and algae living in effluent-receiving streams, and soil invertebrates living in treated farmland or in compost-amended soils, are also exposed to these metabolites. Due to their hormonally active nature, inertness, and lifelong exposure, secondary plant metabolites could be considered a relevant risk factor for humans as well as for a wide range of environmental species.

### 2.2. Classification and Properties of Plant-Derived Metabolites

Secondary metabolites of plants are a heterogeneous family of compounds that are not directly involved in basic metabolic processes (growth, energy metabolism, etc.), but are crucial for plant defense, signaling, and adaptation [1]. Based on their biosynthetic origin, they are generally classified into four main classes (terpenes, phenols, N-containing compounds, and S-containing compounds) of metabolites (Table 1). Terpenes, which are produced by the mevalonate and methylerythritol phosphate (MEP) pathways, play an important role in ecological interactions, including plant–insect signaling, allelopathy, and responses to environmental stress [24]. Monoterpenes such as geraniol, citral, nerol, linalool, and linalyl acetate are volatile compounds that contribute to the aroma of plants and pollinator attraction, and are known to display (anti-)estrogenic or anti-androgenic effects in diverse endocrine assays [25,26]. Diterpenes including steviol, stevioside, and rebaudioside A from the Stevia species have been reported for their anti-progestagenic activity and their hormonal modulatory effect [27]. Triterpenes, such as ginsenoside-Rh2 present in the Panax species, are known for their adaptogenic activity and have been found to show estrogenic action in both in vitro and in vivo studies [28].

The phenolic compounds are derived from the acetate-malonate and shikimic acid pathways; they are involved in various biochemical pathways in the plant cells and play a key role in plant physiology such as protection from harmful UV radiation and pathogens and structural support through lignification [29]. Coumarin is involved in pathogen defense and has been shown to exhibit potent estrogenic activity in hormone receptor assays [30]. Vegetable-borne furanocoumarins, including bergapten, xanthotoxol, angelicin, psoralen, and isopimperatorin, are photoactive compounds of anti-predator defense in plants and exhibit anti-estrogenic and progestagenic activities in some endocrine disruption circuits [31,32,33]. Lignin derivatives, such as bisguaiacol A and bissyringol A, are linked to cell-wall stabilization but have also been reported to induce estrogenic activity and developmental toxicity [34]. Flavonoids are a major subgroup of phenolics, including luteolin and quercetin, which act as antioxidants and UV protectants, and exhibit various hormone activities such as (anti-)estrogenic, anti-androgenic, and anti-progestagenic activities [35,36]. Isoflavonoids such as genistein, equol, and daidzein, found in leguminous plants, are known for their strong phytoestrogenic effect on reproductive and neuroendocrine functions [35,36,37].

The majority of nitrogen-containing secondary metabolites are biosynthesized through the shikimic acid pathway, and they play a role mainly in anti-herbivory and anti-microbial defensive functions [38]. Previous studies suggested that some alkaloids could exhibit estrogenicity in endocrine disruption tests and, as such, could also have an effect on the hormonal system of different organisms [39]. Amygdalin, a cyanogenic glucoside, releases hydrogen cyanide upon hydrolysis when plant tissue is damaged. This substance can be harmful to the plant’s surrounding environment by interfering with hormonal signaling pathways, which may, in turn, affect female reproductive function across organisms, even in herbivores feeding on the plant [40]. Sulfur-containing metabolites formed during sulfur assimilation are involved in various plant defense responses, particularly against pathogen infection and herbivory [41]. Glucosinolates are readily enzymatically cleaved to biologically active compounds (thiocyanates and isothiocyanates), which negatively affect the thyroid gland of animals inhibiting iodine uptake and metabolism [42]. Phytoalexins such as phaseollin and kievitone are biotic stress-inducible antimicrobial compounds and have been found to possess estrogenic and anti-androgenic activities indicating potential involvement in endocrine disruption [43].

Collectively, these facts illustrate the structural diversity and hormonal activity of plant secondary metabolites, some of which have been shown to affect endocrine signaling in human or other mammalian systems. Although our current knowledge is primarily derived from studies involving human or mammalian systems, it is important to note that hormonal pathways are conserved across a broad range of environmental species. This indicates that these metabolites may also affect non-target organisms in the environment.

**Table 1:** Plant-derived secondary metabolites and their reported endocrine-disrupting activities in human or mammalian systems.

Types	Biosynthesis Pathway	Class	Secondary Metabolites	Reported Endocrine Activity	Ref
Terpenes	Mevalonate pathway and methylerythritol 4-phosphate pathway	Monoterpenes	Geraniol, citral, nerol, linalool, and linalyl acetate	(Anti-)estrogenic activity and anti-androgenic activity	[25,26]
		Diterpenes	Steviol, stevioside, and rebaudioside A	Anti-progesterone activity	[27]
		Triterpenes	Ginsenoside-Rh2	Estrogenic activity	[28]
Phenols	Acetate-malonate pathway and shikimic acid pathway	Coumarin	Coumarins	Estrogenic activity	[30]
		Furanocoumarins	Bergapten, xanthotoxol, angelicin, psoralen, and isoimperatorin	Anti-estrogenic activity and progesterone activity	[32,33]
		Lignin	Bisguaiacol A and bissyringol A	Estrogenic activity and developmental toxicity	[34]
		Flavonoids	Luteolin and quercetin	(Anti-)estrogenic activity, anti-androgenic activity, and anti-progesterone activity	[35,36]
		Isoflavonoids	Genistein, equol, and Daidzein	Estrogenic activity and neuroendocrine system	[35,36,37]
N-containing compounds	Shikimic acid pathway	Alkaloids	α-Erythroidin	Estrogenic activity	[39]
		Cyanogenic glucosides	Amygdalin	Indirect female reproductive processes	[40]
S-containing compounds	Sulfur Assimilation	Glucosinolates	Thiocyanate and Isothiocyanates	Negative effects on thyroid function	[42]
		Phytoalexins	Phaseollin and kievitone	Estrogenic activity and anti-androgenic activity	[43]

## 3. Approaches and Models for Environmental Endocrine Disruption Assessment

Understanding the ecological risks of potential EDCs requires a multi-tiered approach encompassing a range of biological systems. These are typically categorized into in silico, in vitro, and in vivo systems, each offering complementary insights into chemical hazard prediction, mechanistic elucidation, and environmentally relevant outcomes (Table 2). This conceptual framework and its practical applications are mainly adopted from recent comprehensive reviews, including Mitchell et al. [44], which details the role of new approach methodologies (NAMs) in the endocrine assessment of fish and amphibians, and the OECD guidance document on standardized testing strategies and regulatory integration for EDCs in aquatic species [44,45].

**Table 2:** Model organisms and platforms for endocrine disruption assessment.

System	Model Organism/ Platform	Target Endpoint	Description	Ref
In silico	QSAR models	Acute and chronic toxicity endpoints in aquatic species	Prediction of aquatic toxicity based on chemical structure and physicochemical descriptors	[46]
	Molecular docking and dynamics simulation	Binding affinity and conformational changes over time	Prediction and validation of binding interactions between chemicals and receptors	[47]
	AOP (Adverse Outcome Pathway)	Molecular initiating events to population-level outcomes	Mechanistic prediction of endocrine disruption and adverse phenotypes	[48]
	HTS data integration	Multi-target bioactivity signatures	Integration of assay data to prioritize and predict endocrine activity	[49,50]
In vitro (Mammalian)	Estrogen and androgen receptor binding affinity	Estrogen receptor (ER) and androgen receptor (AR) binding affinities	Detection of chemical interaction with human ER-α and AR; applicable to estrogenic androgenic hazard screening and prioritization	[51,52]
	Hormone receptor transactivation and yeast ER/AR screen	ER and AR agonistic or antagonistic activities	Stably transfected cell lines or genetically modified *Saccharomyces cerevisiae* expressing human Erα or AR linked to a reporter gene (e.g., luciferase)	[53,54,55,56]
	H295R Steroidogenesis assay	Steroid hormone synthesis (e.g., estradiol, testosterone)	Human adrenocortical carcinoma cell line to detect chemicals that alter the production of steroid hormones	[57]
	Thyroid peroxidase (TPO) inhibition assay	Thyroid peroxidase enzyme inhibition	Detection of chemical interference with TPO activity	[58]
	Transthyretin (TTR) binding assay	Disruption of thyroid hormone transport via TTR	Evaluation of interference with thyroid hormone transport via transthyretin	[59]
In vitro (Non-mammalian)	Fish hormone receptor-based transactivation assay	Hormone receptor activation or inhibition	Recombinant reporter gene assay expressing hormone receptors for endocrine activity screening	[60]
	Primary cell cultures from fish	VTG induction, steroidogenesis, and hormone biosynthesis	Freshly isolated liver or gonadal cells to evaluate endocrine responses	[61,62]
	Amphibian thyroid cell assay	Thyroid hormone disruption	Primary cell culture from amphibians to assess thyroid-disrupting effects	[63]
	Immortalized fish cell lines	Cytotoxicity, estrogenic response, AhR activation, and oxidative stress	Immortalized cell lines for assessing chemical toxicity and endocrine- related responses	[64,65]
	Embryo-based assays	Sex differentiation, thyroid disruption, morphogenesis, and gene expression	Endocrine-disruptive effects with morphological and molecular endpoints	[66,67]
	Fish microsome assay	Enzyme activity related to steroidogenesis and xenobiotic metabolism	Microsomes from fish to assess steroid hormone synthesis and metabolism of EDCs	[68]
	Primary cell cultures from terrestrial invertebrates	Oxidative stress, DNA damage, and hormone-related gene expression	Primary cell cultures for assessing cytotoxicity, oxidative damage, and endocrine-responsive markers	[69,70]
In vivo	Zebrafish (*Danio rerio*)	VTG induction, sex ratio, and reproductive behavior	Multiple endocrine endpoints and reproductive endpoints	[71,72,73,74,75,76,77]
	Rainbow trout (*Oncorhynchus mykiss*)	Plasma VTG levels, hepatic gene expression, and gonad histopathology	Well-established salmonid model in endocrine and reproductive toxicology	[71,75]
	Medaka (*Oryzias latipes*)	Gonadal development and secondary sexual characteristics	Multiple endocrine endpoints and reproductive endpoints	[71,72,73,74,77,78]
	Fathead minnow (*Pimephales promelas*)	Gonad histopathology and reproductive success	Multiple endocrine endpoints and reproductive endpoints	[71,72,74,75,77]
	Daphnia *(Daphnia magna)*	Reproduction rate, molting, and developmental delay	Key aquatic invertebrate for assessing endocrine and reproductive toxicity	[79,80,81]
	Xenopus *(Xenopus laevis)*	Metamorphosis stage, tail resorption, and thyroid gene expression	Amphibian model for thyroid hormone disruption during early development	[82,83,84]
	Stickleback (*Gasterosteus aculeatus*)	Spiggin induction (protein and gene expression)	Detection of androgenic activity via male-specific protein expression in females	[77,85]
	Copepod (*Amphiascus tenuiremis*)	Survival, offspring number, development rate, and sex ratio	Sediment-associated marine invertebrate model for assessing chronic and endocrine toxicity	[86]
	Midge (*Chironomus riparius*)	Emergence timing and gene expression	Sediment-exposed benthic model with endocrine-sensitive endpoints	[87,88]
	New Zealand mud snail (*Potamopyrgus antipodarum*)	Embryo count in brood pouch, survival, and growth	Sediment- and water-associated screening for estrogenic or anti-estrogenic endocrine activity	[89]
	Taxonomy browser (*Lymnaea stagnalis*)	Egg mass production, egg count, survival, growth, and behavior	Assessment of endocrine-related reproductive toxicity and chronic exposure effects	[90]
	Springtail (*Folsomia candida*)	Juvenile production and growth	Soil-dwelling arthropod used for endocrine and developmental effects	[91]
	Earthworm (*Eisenia fetida*)	Cocoon production and survival	Soil organism with reproduction-sensitive endocrine endpoints	[92]
	Honeybee (*Apis mellifera*)	Behavior, learning and reproduction	Pollinator model for assessing sublethal endocrine-disrupting effects	[93,94]

### 3.1. In Silico Models

In silico approaches are now an integral part of the risk assessment of chemical substances at an early stage and enable a rapid and cost-saving screening of huge compound libraries. Quantitative Structure–Activity Relationship (QSAR) models are used to predict the biological activity or toxicity of chemicals based on their molecular structure [46]. These models employ statistical or machine-learning methods and are commonly applied in drug discovery, chemical safety assessments, and environmental toxicology. They help evaluate potential effects of chemicals without the need for experimental testing [46,95]. For structure-based predictions, molecular docking and molecular dynamics simulations evaluate the interaction between chemicals and endocrine targets, such as hormone receptors, and report on binding affinity, stability, and receptor specificity (e.g., AutoDock, SwissDock, GROMACS) [47,96,97]. The Adverse Outcome Pathway (AOP) is a feed-forward framework that provides mechanism-based support by linking molecular initiating events (MIEs) to whole cell responses, organ phenotypes, and population responses. Due to the hierarchical structure of this format, it permits reading across mechanistic data within species and exposure scenarios (OECD AOP Knowledge Base) [48]. Additionally, in vitro high throughput screening (HTS) integration tools, like the US EPA ToxCast and the CompTox Chemicals Dashboard, provide bioactivity profiles for hundreds of molecular targets, enabling prioritization of chemicals, but also supporting trafficability on endocrine pathways [49,50].

### 3.2. In Vitro Models

In vitro systems fill the gap between molecular-level screening and organism-level responses, providing mechanistic insights in controlled and replicable conditions. Although developed in vitro assays are originally utilized to assess potential EDCs in human health contexts, several mammalian in vitro assays have proved substantial value in predicting endocrine-related effects in environmental species [45]. These assays, particularly those focusing on estrogenic [51,53,55], androgenic [52,54,56], and thyroid pathways [58,59], provide mechanistic insights that are often conserved across vertebrate species, thus supporting their application as predictive tools for the potential EDC assessment.

In aquatic toxicology, numerous in vitro-based techniques have been developed with fish species, including receptor-based and tissue-based methodologies. Fish hormone receptor-based transactivation assays utilize cloned hormone receptors from aquatic species expressed in mammalian cells [60]. Reporter constructs containing luciferase under EREs enable quantitation of the activation of the receptors upon treatment with chemicals [98]. In addition to receptor-based assays, primary hepatocytes or gonadal cells isolated from aquatic species are utilized to assess vitellogenin (VTG) induction, steroid hormone synthesis inhibition, and estrogen-responsive gene expression [61,62]. Amphibian thyroid cell assays, especially those derived from Xenopus, serve to evaluate disruption of the hypothalamic–pituitary–thyroid (HPT) axis by monitoring thyroid hormone modulation, typically T3 and T4 [63]. Complementing these techniques, immortalized fish cell lines such as RTgill-W1, ZF4, and RTL-W1 have been applied to assess endocrine response as VTG induction or AhR activation. The RTgill-W1 cell line is among those used for standardized cytotoxicity testing ISO 21115 [64,65]. Embryo-based assays are being increasingly used to further capture endocrine effects during early development. Although embryos are technically whole organisms, they are considered in vitro test systems when used within certain time frames in accordance with regulatory definitions [66,67]. These tests facilitate measurement of VTG expression and thyroid endpoints at vulnerable developmental periods [67]. Notably, in vitro approaches have been extended to terrestrial invertebrates. For instance, earthworm coelomocyte cultures and honeybee fat body or neural cell cultures can be used for studying variance in oxidative stress, DNA damage, immune-like response, and expression of hormone-responsive genes upon soil contaminants or pesticide exposure [69,70]. These models are of specific interest in the context of assessing endocrine risks in soil/pollinator species.

### 3.3. In Vivo Models

In vivo models are considered as the regulatory gold standard for validating endocrine-disrupting effects at the whole-organism level. They provide integrated biological responses encompassing multiple physiological systems. Vertebrates, including zebrafish (*Danio rerio*), rainbow trout (*Oncorhynchus mykiss*), medaka (*Oryzias latipes*), and fathead minnow (*Pimephales promelas*), are commonly used within OECD and USEPA guidelines [71,72,73,74,75]. These fish models are sensitive to critical endocrine apical endpoints such as VTG induction, sex ratio, reproductive success, and histological changes. Therefore, they are widely utilized for academic purposes and governmental testing and are relevant for the risk assessment of potential endocrine-active compounds. In the case of aquatic invertebrates, daphnia (*Daphnia magna*) is considered a sensitive model for evaluating effects on reproduction, molting, and developmental timing under exposure to EDCs [79,80,81]. Xenopus (*Xenopus laevis*) is applied for detecting thyroid hormone disruption during early development by assessing metamorphic stage, tail resorption, and thyroid-related gene expression [82,83]. This model provides sensitive biological endpoints for screening endocrine-disrupting chemicals targeting the thyroid axis. Female stickleback (*Gasterosteus aculeatus*) can be used to assess androgenic activity by the level of spiggin induction. Generally, the spiggin is a male-specific protein and absent in females, indicating a targeted screening method for androgenic agonists [85]. *Amphiascus tenuiremis* serves as a sediment-associated marine model used to evaluate endpoints such as survival, offspring production, development rate, and sex ratio [86].

Midge (*Chironomus riparius*) can provide sediment-relevant insights into endocrine activity through emergence timing and expression of hormone-regulated genes (e.g., ecdysone receptor, juvenile hormone pathway genes) [87,88]. New Zealand mud snail (*Potamopyrgus antipodarum*) and Taxonomy browser (*Lymnaea stagnalis*) can evaluate endocrine-mediated reproductive toxicity under waterborne or sediment exposure. Key endpoints are embryo production, egg mass output, survival, and growth [89,90]. Terrestrial soil-dwelling organisms like springtail (*Folsomia candida*) and earth worm (*Eisenia fetida*) are used to examine reproductive output (e.g., cocoon production and juvenile survival), fertility, and survival in soil, and also have relevance for the assessment of residues of pesticides or veterinary pharmaceuticals [91,92]. Lastly, honeybee (*Apis mellifera*) has been recognized as a valuable model for evaluating sublethal effects of agrochemicals on pollinator behavior, learning, and reproductive-related endpoints [93,94]. Honeybee can also be applied to assess hormonal pathway interference by EDCs [99].

## 4. Endocrine-Disrupting Effects of Plant-Derived Metabolites

Secondary metabolites include a range of bioactive compounds that have the potential to impact endocrine signaling in environmental organisms. Depending on their chemical class, molecular structure, and concentrations within the environment, these metabolites exhibit estrogenic, androgenic, thyroid-disrupting, or steroidogenesis-disrupting effects across vertebrate and invertebrate species. In this section, we synthesize the in vitro and in vivo research on endocrine-disrupting activities of key plant-derived primary metabolite classes and relate their actions to potential ecological implications.

### 4.1. Terpenes

**Figure 1:**
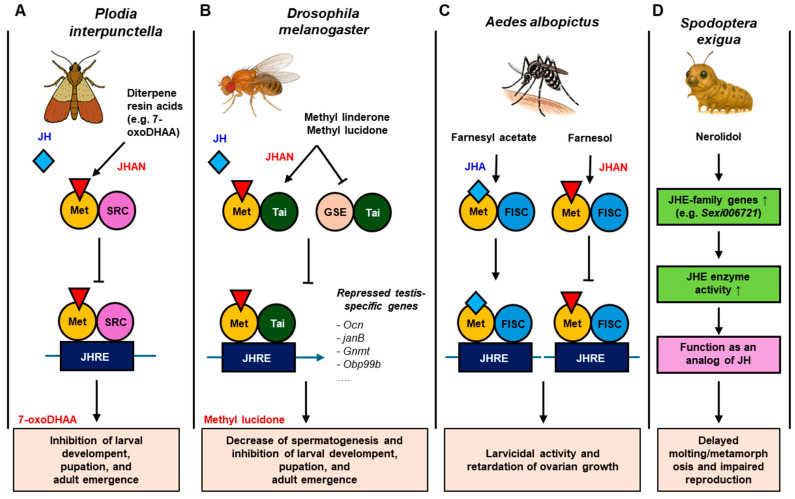
Plant-derived juvenile hormone agonists (JHA) or antagonists (JHNA) disrupt JH signaling pathways in insect species. This figure illustrates how plant-derived secondary metabolites acting as JHA or JHAN interfere with the juvenile hormone (JH) signaling pathways across insect species: *Helicoverpa armigera* (moth), *Drosophila melanogaster* (fruit fly), *Aedes aegypti* (mosquito), and *Spodoptera exigua* (beet armyworm).

Some plant-derived terpenes disrupt endocrine systems of a wide diversity of environmental models by endocrine-related pathways such as juvenile hormone (JH) mediated signaling and estrogen receptor-mediated responses (Figure 1 and Table 3). In invertebrates, disrupted JH-regulated developmental processes were observed by exposure to diterpenoid acids (abietic acid, 7-oxoDHEAA, 7αHDHEAA, dehydroabietic acid, and sandaracopimaric acid), diterpenes (methyl linderone and methyl lucidone), and sesquiterpenes (nerolidol, farnesol, and farnesyl acetate) [100,101,102,103].

JH plays a critical role in regulating insect development, metamorphosis, and reproduction [104]. Both juvenile hormone agonists (JHAs) and antagonists (JHANs) influence these processes by modulating the JH signaling pathway in species-specific ways. In *Helicoverpa armigera*, diterpenoid acids (abietic acid, 7-oxoDHEAA, 7αHDHEAA, dehydroabietic acid, and sandaracopimaric acid) bind to the complex of the methoprene-tolerant (Met) and steroid receptor coactivator (SRC), thereby disrupting a transcriptionally active complex that binds to juvenile hormone response elements (JHREs). Specifically, 7-oxoDHEAA caused inhibition of larval development, pupation, and adult emergence [100]. In *Drosophila melanogaster*, methyl lucidone not only binds to Met in complex with taiman (Tai), but also the complex of germ cell-expressed (GSC) and Tai. This interference was found to affect spermatogenesis and ultimately cause reproductive inhibition and developmental arrest [101]. Conversely, in *Aedes aegypti*, farnesyl acetate activates the Met with the βFtz-F1-interacting steroid receptor coactivator (FISC) complex and stimulates JHRE transcription, mimicking natural JH signaling. However, farnesol binds to the complex of Met-FISC and represses the transcription. Both exposures result in larvicidal activity and suppression of ovarian development, illustrating the potential of JHAs for mosquito control [102]. In *Spodoptera exigua*, nerolidol exposure caused upregulation of juvenile hormone esterase (JHE)-family genes (e.g., Sexi006721) and increased JHE enzyme activity. This leads to delayed molting and impaired reproduction [103]. Although these phenotypic effects were investigated in pests, the mechanisms controlling them via JH signaling are commonly conserved among insect taxa. Therefore, these compounds may exert similar endocrine-disrupting effects in other non-target insects, which is also a cause for concern regarding potential wide-ranging implications in invertebrate communities.

Similarly, diterpenes and triterpenes affect the endocrine system in aquatic vertebrates. Bakuchiol induced estrogenic activity in medaka liver, indicated by enhanced GFP expression in the presence of an estrogen-responsive reporter [105]. Abietic acid, dehydroabietic acid and β-sitosterol induced changes in VTG expression (liver and plasma) and metabolized enzyme activity in rainbow trout and zebrafish [106,107,108]. In vivo exposure to betulinol in zebrafish resulted in a significant reduction in plasma VTG levels in F_0_ females, while F_1_ males exhibited elevated VTG concentrations. Additionally, betulinol treatment stimulated spawning activity. Histological analysis of the gonads, along with sex hormone profiling, indicated notable effects on reproductive physiology [107]. Gibberellic acid, when tested on zebrafish, caused inhibition of organogenesis to be followed by oxidative stress and an increase in the expression of hormone-responsive genes [109]. Curiously, both abietic acid and dehydroabietic acid exhibited endocrine-disrupting effects in invertebrate and vertebrate models. Overall, these findings highlight the endocrine-disrupting potential of naturally occurring terpenes in ecologically relevant species.

**Table 3:** Summary of plant-derived terpenes affecting endocrine function in environmental models.

Metabolite	System	Model Organism/Platform	Target Endpoint	Effect	Ref
7-oxoDHAA	In vitro/ In vivo	Yeast two-hybrid/ *Plodia interpunctella*	JH-mediated metamorphosis, pupation, and adult emergence	Disruption of JH receptor complex formation; inhibition of larval growth, pupation, and adult emergence	[100]
7α-HDHAA	In vitro/ In vivo	Yeast two-hybrid/ *Plodia interpunctella*	JH-mediated metamorphosis, pupation, and adult emergence	Inhibition of Met-SRC receptor binding	[100]
Abietic acid	In vivo	Rainbow trout (*Oncorhynchus mykiss*)	Vitellogenin gene expression	Slight induction of estrogenic response via oral exposure through feed mixture (abietic acid 37% and dehydroabietic acid 6%)	[106]
	In vitro/ In vivo	Yeast two-hybrid/ *Plodia interpunctella*	JH-mediated metamorphosis, pupation, and adult emergence	Presence of JHAN activity; manifestation of anti-feedant activity without developmental toxicity	[100]
Bakuchiol	In vivo	Medaka (*Oryzias melastigma)*	Liver-based reporter of estrogenic activity	Elevation of GFP fluorescence in medaka liver as an indication of estrogenic activity	[105]
Betulinol	In vivo	Zebrafish (*Danio rerio*)	Plasma vitellogenin, sex hormones, gonad histology, and reproduction	Reduction in VTG in F_0_ females; elevation of VTG in F_1_ males; stimulation of spawning	[107]
Dehydroabietic acid	In vitro/ In vivo	Yeast two-hybrid/ *Plodia interpunctella*	JH-mediated metamorphosis, pupation, and adult emergence	Moderate JH receptor binding interference	[100]
	In vivo	Rainbow trout (*Oncorhynchus mykiss*)	Hepatic enzyme activity (EROD, GST), plasma vitellogenin, and E2 response	Modulation of metabolic enzyme activity; attenuation of estradiol-induced vitellogenin synthesis	[108]
		Zebrafish (*Danio rerio*)	Plasma vitellogenin, sex hormones, gonad histology, and reproduction	Reduction in plasma vitellogenin in F_0_ males; alteration of gonadal development	[107]
Farnesol	In vivo	Mosquito (*Aedes albopictus*)	JH signaling disruption, gene expression, and ovarian development	JH antagonist activity; retardation of ovarian growth	[102]
Farnesyl acetate	In vivo	Mosquito (*Aedes albopictus*)	JH signaling disruption, gene expression, and ovarian development	JH agonist activity; retardation of ovarian growth	[102]
Gibberellic acid	In vivo	Zebrafish embryos (*Danio rerio*)	Developmental toxicity (heart, liver, eye, and kidney) and oxidative stress	Inhibition of organogenesis; alteration of gene expression (e.g., Myl7, Vmhc, Fabp10a, Kim1); elevation of ROS	[109]
Methyl linderone	In vitro/ In vivo	Yeast two-hybrid/*Drosophila melanogaster*	JH receptor complex (Met-Taiman, GCE-Taiman) and larval-pupal development	Moderate inhibition of JH receptor dimerization and developmental interference; weaker than methyl lucidone	[101]
		Yeast two-hybrid/ *Plodia interpunctella*	JH-mediated metamorphosis, pupation, and adult emergence	Potent JHAN activity	[100]
Methyl lucidone	In vitro/ In vivo	Yeast two-hybrid/*Drosophila melanogaster*	JH receptor complex (Met-Taiman, GCE-Taiman) and larval-pupal development	Inhibition of JH-mediated receptor dimerization and gene expression; suppression of larval development	[101]
Nerolidol	In vivo	Beet armyworm (*Spodoptera exigua*)	Juvenile hormone esterase (JHE) gene expression, JH titer, and JHE enzyme activity	Elevation of JH titer and JHE activity; induction of developmental disruption and fecundity reduction	[103]
Sandaracopimaric acid	In vitro/ In vivo	Yeast two-hybrid/ *Plodia interpunctella*	JH-mediated metamorphosis, pupation, and adult emergence	Receptor-binding disruption	[100]
β-sitosterol	In vivo	Rainbow trout (*Oncorhynchus mykiss*)	Vitellogenin gene expression	Robust upregulation of VTG expression as an indication of estrogenic activity	[106]

### 4.2. Flavonoids

A diverse range of flavonoids with endocrine-disrupting potential were also iden-tified in aquatic vertebrate models, including flavonoids acting through inhibition of the aromatase enzyme, activation of estrogen receptor, and induction of VTG (Table 4). In vitro assays using ovarian microsomes from rainbow trout demonstrated that several flavonoids, including 7,4′-dihydroxyflavone, α-naphthoflavone, apigenin, DL-aminoglutethimide, equol, flavone, and quercetin, potently inhibited aromatase activity [110]. Conversely, genistein and biochanin A exhibited relatively weak inhibitory activity when compared to flavone [110]. In the hepatic microsome assay conducted on Nile tilapia (*Oreochromis niloticus*), chrysin and quercetin exhibited aromatase activity [111]. Specifically, chrysin was observed to be more potent than quercetin. In vivo exposure studies further substantiated modulation of estrogenic pathways [112]. A fish diet containing high levels of genistein and daidzein resulted in the induction of VTG expression in male goldfish (*Carassius auratus*) [112]. In particular, their exposure influenced sex-related gene expression in Russian sturgeon (*Acipenser gueldenstaedtii*) [113]. Although genistein and daidzein induced significant upregulation of *cyp19*, *vtg*, and *vasa* in the brain of intersex individuals, genistein alone caused a downregulation of *erα* and *erβ* but elevated VTG, implying complex or non-receptor-mediated estrogenic effects. In gonads, they caused overall downregulation of sex-related genes (e.g., *dmrt1*, *amh*, *sox9*), compared to the control. They also exhibited upregulation of *vtg*, *erα*, and *erβ* in the liver [113].

Other flavonoids also presented estrogenic activity in the transgenic zebrafish expressing tissue-specific GFP under estrogen-responsive promoters (ERα and ERβ) [36]. Gequol, liquiritigenin, isobavachin, and phenoxodiol induced the expression of GFP in both the liver and heart, indicating ER signaling and tissue- and dose-dependent variability. While isobavachin and phenoxodiol elicited responses only at the highest concentration (1000 nM) in the heart and liver, gequol and liquiritigenin produced dose-dependent effects [36]. Meanwhile, tannic acid was evaluated in plateau pika (*Ochotona curzoniae*) and root vole (*Microtus oeconomus*) models, showing increased plasma androgen and estrogen levels without significant alteration in GnRH expression [114]. Together, these findings illustrate the diverse endocrine activities of flavonoids, which act via multiple pathways including estrogen receptor agonism, aromatase inhibition, and modulation of sex-related gene networks.

**Table 4:** Summary of plant-derived flavonoids affecting endocrine function in environmental vertebrate and invertebrate models.

Metabolite	System	Model Organism/Platform	Target Endpoint	Effect	Ref
7,4′-dihydroxyflavone	In vitro	Ovarian microsomes (*Oncorhynchus mykiss*)	Aromatase enzyme inhibition	Potent inhibition, relative potency: 3.7 (vs. flavone = 1.0)	[110]
α-naphthoflavone	In vitro	Ovarian microsomes (*Oncorhynchus mykiss*)	Aromatase enzyme inhibition	Potent inhibition, relative potency: 3.2 (vs. flavone = 1.0)	[110]
Apigenin	In vitro	Ovarian microsomes (*Oncorhynchus mykiss*)	Aromatase enzyme inhibition	Potent inhibition, relative potency: 8.7 (vs. flavone = 1.0)	[110]
Biochanin A	In vitro	Ovarian microsomes (*Oncorhynchus mykiss*)	Aromatase enzyme inhibition	Weak inhibition, relative potency: <0.3 (vs. flavone = 1.0)	[110]
Chrysin	In vitro	Hepatic microsomes (*Oreochromis niloticus*)	Aromatase enzyme inhibition	Potent anti-aromatase effect	[111]
Daidzein	In vivo	Male goldfish (*Carassius auratus*)	Plasma vitellogenin	VTG induction in a fish diet containing high levels of genistein and daidzein	[112]
		Russian sturgeon (*Acipenser gueldenstaedtii*)	Expression of sex-related genes (amh, ar, cyp19, dmrt1, erα, erβ, foxl2, sox9, star, vasa, and vtg)	Pronounced downregulation of genes in liver and gonads; endocrine disruption pattern	[113]
DL-aminoglute- thimide	In vitro	Ovarian microsomes (*Oncorhynchus mykiss*)	Aromatase enzyme inhibition	Potent inhibition, relative potency: 19 (vs. flavone = 1.0)	[110]
Equol	In vitro	Ovarian microsomes (*Oncorhynchus mykiss*)	Aromatase enzyme inhibition	Potent inhibition, relative potency: 0.9 (vs. flavone = 1.0)	[110]
	In vivo	Japanese medaka (*Oryzias latipes*)	Gonadal development, secondary sex characteristics	Impaired spermatogenesis, fibrosis, altered female oocyte development, and sex reversal of external characteristics	[115]
Flavone	In vitro	Ovarian microsomes (*Oncorhynchus mykiss*)	Aromatase enzyme inhibition	Potent inhibition	[110]
Genistein	In vitro	Ovarian microsomes (*Oncorhynchus mykiss*)	Aromatase enzyme inhibition	Weak inhibition, relative potency: <0.2 (vs. flavone = 1.0)	[110]
	In vivo	Russian sturgeon (*Acipenser gueldenstaedtii*)	Expression of sex-related genes (amh, ar, cyp19, dmrt1, erα, erβ, foxl2, sox9, star, vasa, and vtg)	Alteration of gene expression in all tissues, with moderate induction of feminization-related markers	[113]
		Male goldfish (*Carassius auratus*)	Plasma vitellogenin	VTG induction in a fish diet containing high levels of genistein and daidzein	[112]
	In vivo	Japanese medaka (*Oryzias latipes*)	Gonadal development, secondary sex characteristics	Delayed oocyte maturation, increased oocyte atresia, ovarian fibrosis, and disrupted sex phenotype expression	[115]
Gequol (S-equol)	In vivo	Zebrafish (*Danio rerio*)	Estrogenic tissue-specific GFP response	Dose-dependent GFP expression in liver (ER-alpha) and heart (ER-beta)	[36]
Isobavachin	In vivo	Zebrafish (*Danio rerio*)	Estrogenic tissue-specific GFP response	Dose-dependent GFP expression in liver (ER-alpha) and heart (ER-beta)	[36]
Liquiritigenin	In vivo	Zebrafish (*Danio rerio*)	Estrogenic tissue-specific GFP response	GFP expression at 1000 nM in heart (ER-beta)	[36]
Phenoxodiol	In vivo	Zebrafish (*Danio rerio*)	Estrogenic tissue-specific GFP response	GFP expression at 1000 nM in liver (ER-alpha) and heart (ER-beta)	[36]
Quercetin	In vitro	Hepatic microsomes (*Oreochromis niloticus*)	Aromatase enzyme inhibition	Weak anti-aromatase effect compared to chrysin	[111]
	In vitro	Ovarian microsomes (*Oncorhynchus mykiss*)	Aromatase enzyme inhibition	Potent inhibition, relative potency: 5.3 (vs. flavone = 1.0)	[110]
Tannic acid	In vivo	Plateau pika (*Ochotona curzoniae*)/Root vole (*Microtus oeconomus*)	GnRH and plasma androgen and estrogen levels	Elevation of plasma androgen and estrogen without significant change in GnRH.	[114]

### 4.3. Other Secondary Metabolites

Other plant-derived metabolites, such as coumarins and alkaloids, exhibited endocrine-disrupting effects in aquatic species, acting through gene regulation, hormone synthesis, and metabolic interference (Table 5). Coumestrol exposure in Russian sturgeon caused the modulation of multiple sex-related genes, including *amh*, *ar*, *cyp19*, *dmrt1*, and *vtg* [113]. Overall gene expression in gonads was suppressed compared to controls. Although the influence was mild when compared to genistein and daidzein, coumestrol exhibited interference with sex differentiation pathways. Another coumarin, scopoletin, also showed the disruption of hormone-related metabolic profiles in zebrafish embryos [116]. Embryos exposed to higher concentrations (particularly 20 μM) exhibited delayed hatching, morphological deformities, and reduced locomotor activity, indicating overt developmental toxicity. Pathway enrichment analysis demonstrated that scopoletin disrupted several critical metabolic pathways (glutathione metabolism, purine metabolism, citrate (TCA) cycle, and arginine and proline metabolism). In the alkaloid category, 3,3′-diindolylmethane (DIM) and indole-3-carbinol (I3C) both induced VTG and CYP1A gene expression in the liver of rainbow trout, suggesting the activation of both estrogenic and xenobiotic metabolism pathways [117]. Interestingly, caffeine exhibited a reduction in plasma 17β-estradiol (E2) levels, hepatic VTG gene expression, and induced histological changes in the liver and testes of yellow-tail tetra (*Astyanax altiparanae*) [118]. Furthermore, nicotine exposure in zebrafish resulted in the widespread suppression of endocrine-related biomarker genes, including *vtg1*, *cyp19a1a*, and *cyp19a1b*, consistent with anti-estrogenic effects [119]. These outcomes indicate suppression of estrogen signaling and reproductive impairment, raising concerns about environmentally relevant concentrations of caffeine and nicotine in source water [120,121]. Lastly, reserpine, a known neuroactive alkaloid, caused developmental toxicity, thyroid dysfunction via HPT axis disruption, and dysregulation of endocrine-related genes in zebrafish embryos [122].

Taken together, these findings confirm that plant-derived metabolites possess hormonal bioactivity capable of modulating endocrine and reproductive functions in environmental organisms, necessitating further investigation into their environmental occurrence, mechanisms of action, and long-term ecological implications.

**Table 5:** Summary of plant-derived coumarins and alkaloids affecting endocrine function in environmental vertebrate and invertebrate models.

Metabolite	System	Model Organism/Platform	Target Endpoint	Effect	Ref
Coumestrol	In vivo	Russian sturgeon (*Acipenser gueldenstaedtii*)	Expression of sex-related genes (amh, ar, cyp19, dmrt1, erα, erβ, foxl2, sox9, star, vasa, and vtg)	Mild modulation of gene expression	[113]
Scopoletin	In vivo	Zebrafish (*Danio rerio*)	Metabolic profiles in embryos	Disruption of hormone-relevant metabolic pathways; indirect endocrine disturbance potential	[116]
3,3′-Diindolylmethane (DIM)	In vivo	Rainbow trout (*Oncorhynchus mykiss*)	Hepatocarcinogenesis and gene expression of vitellogenin and CYP1A	VTG and CYP1A Induction	[117]
Caffeine	In vivo	Yellow-tail tetra (*Astyanax altiparanae*)	Plasma steroid levels (Testosterone, 11-KT), hepatic vitellogenin gene expression, testis and liver histology	Reduction in E2 concentration; reduction in VTG gene expression	[118]
Indole-3-carbinol (I3C)	In vivo	Rainbow trout (*Oncorhynchus mykiss*)	Hepatocarcinogenesis and gene expression of vitellogenin and CYP1A	VTG and CYP1A induction	[117]
Nicotine	In vivo	Zebrafish (*Danio rerio*)	Endocrine biomarker gene expression (vtg1, vtg2, cyp19a1a, and cyp19a1b)	Significant downregulation of all endocrine biomarker genes	[119]
Reserpine	In vivo	Zebrafish (*Danio rerio*)	CNS neuron differentiation, thyroid development, locomotion, and gene expression	Developmental toxicity; thyroid dysfunction via HPT axis disruption; dysregulation of endocrine related genes	[122]

### 4.4. Ecological Implications of Endocrine Disruption Driven by Plant-Derived Secondary Metabolites

In the previous section, we confirmed that plant-derived secondary metabolites can induce adverse endocrine-disrupting effects on environmental species, ranging from the molecular to the individual organismal level. These findings raise concerns that chronic exposure can also lead to consequences at the ecological level, affecting population dynamics and community structure [123,124,125]. It has been reported that natural and synthetic endocrine disruptors such as E2, 17α-ethinylestradiol (EE2), and polychlorinated biphenyls (PCBs), once released or accumulated in environmental compartments, result in reproductive failure, alter population dynamics, and disrupt aquatic community structure [126,127,128,129]. However, to the best of our knowledge, there are no specific studies that have demonstrated the impact of phytoestrogens or other plant-derived secondary metabolites on population dynamics or ecological community. Nevertheless, some studies have shown that chronic exposure to low environmental concentrations of phytoestrogens, such as genistein and equol, can induce endocrine-disrupting effects in aquatic species, including VTG induction, altered sex hormone levels, and reduced reproductive success [107,115]. Kiparissis et al. [115] concluded that isoflavone compounds should be considered candidate estrogenic compounds that may be involved in the alteration of sexual development in feral fish populations. Chronic low-dose (100-day) exposure to genistein (up to 1000 µg/L) and equol (0.4–0.8 µg/L) disrupted gonadal development in Japanese medaka. Equol induced testis–ova in 87% of males at 0.8 µg/L, and both compounds caused oocyte atresia, delayed maturation, and stromal proliferation in females. These results demonstrate that environmentally relevant concentrations of isoflavones can strongly interfere with sexual differentiation in fish. Christianson-Heiska et al. [107] conducted a two-generation zebrafish study with dehydroabietic acid, betulinol, and gibberellic acid. The results confirmed that these compounds can disrupt reproductive physiology through altered vitellogenin levels, impaired gametogenesis, and changes in sex steroid profiles. Notably, F0 males exhibited disrupted spermatogenesis, while F1 females showed reduced vitellogenic oocytes, indicating generation-specific sensitivity. Despite histological alterations, reproductive output was not suppressed, suggesting potential compensatory mechanisms [107].

In addition, Blazer et al. [130] investigated the reproductive health indicators of fish across three major river basins in Pennsylvania (Delaware, Susquehanna, and Ohio), focusing on biomarkers such as testicular oocytes (TO) and plasma VTG levels. Among the measured chemical analytes, estrone was the only hormone that showed a statistically significant correlation with TO prevalence and severity. Furthermore, a higher percentage of agricultural land use in the watershed was also significantly associated with increased TO prevalence and severity (*r* ≈ 0.7, *p* < 0.02). Notably, plant-derived sterols such as β-sitosterol and stigmasterol were detected in sediment samples not only at heavily impacted locations but also at less affected sites [130]. β-sitosterol has been reported to have the estrogenic activity in rainbow trout [106]. These results suggest that non-point source pollution from agricultural activities may substantially contribute to endocrine disruption in fish. Moreover, the widespread detection of phytosterols indicates that they may act as indirect contributors of reproductive effects in aquatic organisms.

Taken together, these findings highlight the need to move beyond single-biomarker toxicological or individual assessments. Understanding the prolonged influence of natural endocrine-active substances requires an integrated ecological approach that considers population dynamics, biological diversity, and ecosystem functionality.

## 5. Future Perspectives

In this review, we have confirmed the potential of various plant secondary metabolites to function as endocrine disruptors in environmental organisms, highlighting their significance in environmental toxicology. A significant number of previous studies on plant-derived metabolites have been conducted on humans and mammalian model species, with fewer focused on environmental organisms. Notably, some secondary metabolites have been shown to induce endocrine disruption not just in one species, but across a range of species including humans, mammals, fish, and insects. For instance, abietic acid demonstrated estrogenic activity in human T-47D cells and mild estrogenic effects when mixed with feed for rainbow trout. Additionally, it exhibited JHAN activity in an in vitro system [100,106]. These cross-species endocrine activities indicate that metabolites originally investigated in the context of the mammalian endocrine system may also affect hormonal signaling in a variety of environmental species, which therefore leads to general ecological risks. Many monitoring studies have reported that concentrations of metabolites fall within the potential exposure range in the environment and can also impact wastewater ecology. These findings suggest that environmental species may be chronically exposed to these metabolites as well. Therefore, systematic research into environmental exposure routes and consistent monitoring of these potential secondary metabolites are crucial for understanding their ecological impact and guiding regulatory responses.

Building on this, our review provides valuable insights into the potential adverse impacts of these natural compounds, particularly since natural compounds are becoming more abundant in the environment. We emphasize the need for extensive assessment efforts targeting non-mammalian environmental models, which remain underexplored in current research. Comprehensive initial studies focused on environmental organisms are essential for the systematic evaluation of candidate secondary metabolites, utilizing in silico approaches such as molecular docking, QSAR modeling, and HTS. Potential metabolites identified through in silico approaches should be validated through targeted in vitro and in vivo studies with relevant environmental models. This multilevel strategy will enable a thorough assessment of the ecological effects of plant-derived EDCs and help us understand their environmental risks.

## 6. Conclusions

This review highlights the potential of various plant-derived secondary metabolites (flavonoids, terpenes, alkaloids, coumarins, and other classes) to act as EDCs in environmental organisms, emphasizing their significance in environmental toxicology. These metabolites have gained considerable attention in both plant and human research due to their bioactivity and function. However, their persistence and endocrinal activities suggest that they may also represent a new class of potential environmental endocrine disruptors, posing emerging ecological risks. The presence of these metabolites in surface waters, soils, and sediments may result in the chronic, low-dose exposure of aquatic and terrestrial organisms, potentially leading to the disruption of hormonal processes associated with reproduction, development, and metabolism. Notably, certain metabolites showed conserved endocrine-disrupting activities across multiple species, indicating that they have a wider ecological significance. These findings emphasize the need for regulatory frameworks that incorporate naturally occurring compounds into endocrine risk assessments. Given the ecological risks associated with plant-derived secondary metabolites, it is essential to conduct thorough investigations on underexplored environmental species using international standard test methods. A tiered approach, combined with targeted in vitro and in vivo validations, provides a practical way forward. Ultimately, bridging toxicological insights with ecological relevance will be critical to accurately characterize the endocrine-disrupting activity of plant-derived metabolites in natural ecosystems.

## Data Availability

No new data were created or analyzed in this study.
